# A machine learning-based risk prediction model for Hospitalized patients with deep vein thrombosis

**DOI:** 10.7717/peerj.21524

**Published:** 2026-07-30

**Authors:** Xue Wang, Xiankai Chen, Jun Mao, Meiling Liu, Leping Yan, Wuping Sun, Jingjie Song

**Affiliations:** Department of Clinical Laboratory, The Fifth Affiliated Hospital of Southern Medical University, Guangzhou, China

**Keywords:** Deep vein thrombosis, Machine learning, SHAP, D-dimer

## Abstract

**Background:**

Deep vein thrombosis (DVT) is a common thrombotic condition with substantial morbidity when not identified early. Machine learning (ML)–based predictive models may improve early identification of patients at high risk for DVT, but few clinically applicable early-risk models exist.

**Objectives:**

To develop and internally validate a ML model using routinely available clinical and laboratory indicators for early risk prediction of DVT, and to identify the most influential predictors using model explainability techniques.

**Methods:**

We retrospectively analyzed clinical data from 231 patients evaluated at the Fifth Affiliated Hospital of Southern Medical University between January 2017 and June 2024. Patients were labeled as DVT occurrence (*n* = 159) or non-occurrence (*n* = 72). Seven candidate predictors were selected by Least Absolute Shrinkage and Selection Operator (LASSO) regression. The dataset was split into training (70%, *n* = 162) and test (30%, *n* = 69) sets. Five ML algorithms were trained: XGBoost, CatBoost, Random Forest (RF), Logistic Regression, and Support Vector Machine, with hyperparameter tuning on the training set. Model performance was assessed by 5-fold cross-validation and on the held-out test set using Area Under the Receiver Operating Characteristic Curve (AUC), accuracy, recall, and F1 score. The best model was further interpreted via feature importance and Shapley Additive Explanations (SHAP).

**Results:**

LASSO selected seven predictors: hemoglobin, platelet count, leukocyte count, fibrinogen, prothrombin time, D-dimer (DD), and glucose. The Random Forest model showed the best discrimination (test-set AUC = 0.874), with favorable accuracy, recall, and F1 compared with other classifiers (detailed metrics reported in the manuscript). In the RF model, D-dimer had the highest feature-importance contribution; SHAP analysis confirmed DD as the dominant risk driver and characterized the directions and relative effects of other features.

**Conclusions:**

We developed an internally validated ML model for early DVT risk prediction using seven routine clinical variables; Random Forest achieved the best performance and identified D-dimer as the most influential predictor. This model may support earlier identification and intervention for patients at risk of DVT, pending external validation and prospective evaluation.

## Introduction

Deep vein thrombosis (DVT) is a prevalent thrombotic disorder that primarily affects the deep veins of the lower extremities (Geerts et al., 2004). Its pathophysiological mechanisms include blood stasis, vascular endothelial injury, and hypercoagulability (Ji et al., 2022; Nahm, 2022; Yan et al., 2021). The global incidence of DVT is estimated to be approximately 1–2 cases per 1,000 individuals, with risk factors such as advancing age, postoperative status, malignancy, and prolonged immobility. DVT can lead to serious complications, including pulmonary embolism (PE). Early diagnosis and treatment are essential to prevent adverse outcomes (Robertson et al., 2021).

In recent years, machine learning (ML) algorithms(Deo, 2015), such as Limit Gradient Boost (XGBoost) (Chen & Jiang, 2022), logistic regression (LR) (Song et al., 2021), Categorical Boost (CatBoost) (Hancock & Khoshgoftaar, 2020), Random Forest (RF) (Hu & Szymczak, 2023; Shi et al., 2023), and support vector machine (SVM) (Huang et al., 2018), have increasingly assumed a pivotal role in the risk prediction of diseases and its associated complications. As the medical field transitions from traditional models to precision medicine paradigms, ML has emerged as a critical technology for disease discovery, diagnosis, and treatment. The robust data processing and analytical capabilities inherent in ML underscore its significant potential within healthcare. Currently, DVT diagnosis predominantly relies on imaging techniques, with venography historically recognized as the “gold standard” (Kearon, 2016). However, this invasive detection method presents certain limitations that may lead to adverse effects such as allergic reactions to contrast media and renal toxicity (Cho, 2015). Consequently, employing ML to identify easily detectable biomarkers with high accuracy and sensitivity may be useful for clinical prevention, early diagnosis, management of DVT, and clinical decision-making. Although not a replacement for imaging, ML-based approaches may provide scalable and efficient tools to complement diagnostic methods.

Consequently, this study held relevance for improving identification of high-risk individuals and may contribute to more timely clinical decision-making. Although some studies have utilized ML to establish VTE risk prediction models (Hou et al., 2023; Ryan et al., 2021; Xu et al., 2023), including those for first-onset VTE (DVT and PE) during admission, comparative analyses among different ML models remain limited.

In recent years, machine learning (ML) algorithms have played an increasingly important role in disease risk prediction. At present, some studies have used ML to establish VTE and DVT risk prediction models, but comparative studies of different ML models in early warning for hospitalized patients are still limited. Therefore, this study aimed to develop and validate an early risk prediction model for DVT using five ML models based on routine clinical and laboratory indicators, so as to identify key risk factors and provide a reference for individualized prevention and early intervention.

Several ML-based models have been developed for venous thromboembolism and deep vein thrombosis risk prediction. However, most existing models lack comprehensive comparison of different algorithms, and few models focus on early risk stratification using routine laboratory indicators for hospitalized patients. Therefore, our study constructed and compared five machine learning models to fill this gap.

This study aimed to develop a risk prediction model identify patients at high risk of developing DVT based on early clinical data utilizing five ML models: XGBoost, LR, CatBoost, RF, and SVM. Through these ML models, we aimed to identify early predictive features associated with DVT risk, thereby supporting more individualized risk stratification and informing potential prevention strategies. Consequently, this study held significant importance for the early risk identification and intervention of hospitalized patients at risk of DVT, contributing to mitigation of its serious consequences.

## Materials and Methods

### Study population

**Figure 1:**
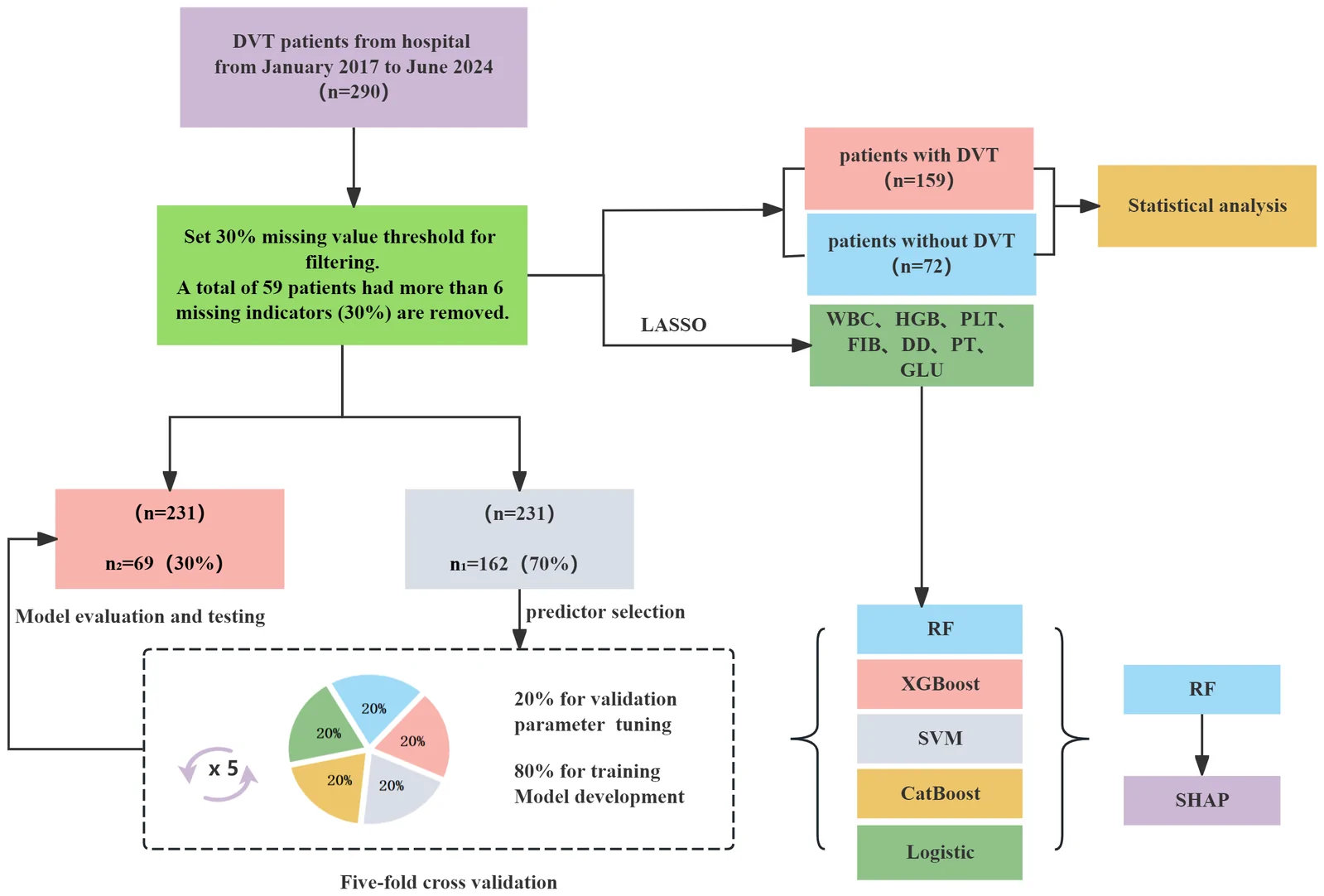
Flowchart of study design, data processing and machine learning model development for deep vein thrombosis risk prediction. This flowchart illustrates the complete analytical workflow of the study. A total of 290 patients with deep vein thrombosis (DVT) admitted to the hospital from January 2017 to June 2024 were initially enrolled. A 30% missing value threshold was set for data filtering, and 59 patients with an indicator missing rate exceeding 30% were excluded, resulting in a final study cohort of 231 subjects, including 159 DVT patients and 72 non-DVT control subjects. The least absolute shrinkage and selection operator (LASSO) regression was used to select core predictive variables from 7 candidate clinical and laboratory indicators, including white blood cell count (WBC), hemoglobin (HGB), platelet count (PLT), fibrinogen (FIB), D-dimer (DD), prothrombin time (PT), and fasting blood glucose (GLU). The dataset was split with a five-fold cross-validation strategy: 80% of the samples were used for model training and parameter tuning, and 20% were used for model validation in each fold, with the cross-validation process repeated 5 times. Five machine learning models were constructed based on the selected predictors, including Random Forest (RF), Extreme Gradient Boosting (XGBoost), Logistic Regression, Categorical Boosting (CatBoost), and Support Vector Machine (SVM). SHapley Additive exPlanations (SHAP) was applied to interpret the feature importance of the optimal model, and model performance evaluation and statistical analysis were finally performed.

The workflow of this study is shown in Fig. 1. This retrospective study included 290 patients with VTE who were admitted to the Fifth Affiliated Hospital of Southern Medical University between January 2017 and June 2024. Data were collected from the electronic medical record (EMR) system of our hospital. History of DVT was determined through electronic medical record chart review. The diagnosis of VTE was based on the Guidelines for the Prevention and Treatment of Thrombotic Diseases established by the Chinese Medical Association. Inclusion criteria were patients aged 18–90 years with no prior deep vein thrombosis (DVT) before admission. Exclusion criteria were previous DVT, regular postoperative follow-up, and incomplete or missing electronic medical records or follow-up data. This study was approved by the Ethics Committee of the Fifth Affiliated Hospital of Southern Medical University (2024-JYYXK-K-003) and conducted in accordance with relevant regulations. Informed consent was waived due to the retrospective design, minimal risk, and full data anonymization.

### Data preprocessing and feature selection

A threshold of 30% for missing values was established. Among the 18 indicators collected, patients with more than six missing indicators were excluded, resulting in the exclusion of 59 patients. Ultimately, patients were categorized into a DVT group (*n* = 159) and a control group without DVT (*n* = 72). 70% (*n* = 162) of the data were utilized for model training while 30% (*n* = 69) as a tested. Missing data was missing at random (MAR). Multiple imputation was performed using the mice package in R with five chains, and the missing patterns were visualized using the VIM package (Zhang, 2015). This imputation covered all seven key features selected by LASSO regression, including hemoglobin (HGB), platelets (PLT), leukocytes (WBC), fibrinogen (FIB), prothrombin time (PT), D-dimer (DD), and glucose (GLU). The distribution of missing data is shown in Fig. S1. Lasso regression (Wang et al., 2022a) was then used to identify significant feature variables with lambda.lse selected as the final variance screening criteria.

### Performance and validation of models

Five ML models were developed: SVM, LR, XGBoost, RF and CatBoost. A total of 231 clinical samples were split into a training set (*n* = 162) and a test set (*n* = 69) based on a 7:3 ratio. 5-fold cross-validation was performed on the training set. To ensure robust evaluation, each model was evaluated using 5-fold cross-validation repeated 1000 times within the training set, and the 95% confidence intervals of performance metrics were estimated using the 2.5th and 97.5th percentiles of the resulting empirical distributions. Overall, the RF model performed the best on the testing set.The predictive efficacy of each model was then verified against the testing dataset, using receiver operating characteristic (ROC) (Nahm, 2022) curves to illustrate differences in AUC values across models.

The XGBoost, LR, RF, CatBoost, and SVM models were implemented using the default hyperparameter settings of the corresponding R packages unless otherwise specified. Model performance was evaluated using repeated 5-fold cross-validation on the training set.

### Feature rankings in the optimal model

Based on model evaluation results, we selected the best-performing ML model. Feature importance analysis was conducted to assess the impact of features on predictions, with importance calculated according to the model’s mechanisms. We analyzed seven key features from the top-performing RF model, visualizing their significance with importance ranking. Additionally, Shapley Additive Explanation(SHAP) was used to interpret predictions (Lundberg & Lee, 2017), illustrating how each feature contributed to the direction and magnitude of the results.

LASSO regression was used for feature selection. After model construction, feature importance was used to rank the selected variables at the global level, while SHAP values were used to interpret the direction and magnitude of each feature’s contribution to the model predictions.

### Statistical processing

Statistical analysis was performed using R version 4.1.2. The Shapiro-Wilk test was used to evaluate the normality of measurement data. Since data were not normally distributed, they were presented as median (interquartile range), and the Wilcoxon rank sum test was used for group comparisons. Categorical data were presented as counts and percentages (%), with comparisons conducted by Pearson’s Chi-squared test (Chang, Li & Qi, 2023). A significant level of α = 0.05 was applied.

## Results

### Patient characteristics

231 patients were included in the final cohort for this study. The LASSO regularization process resulted in 7 potential predictors on the basis of 162 patients in the training data set, which were used for model development (Figs. S2, S3). The baseline characteristics of patients in the two groups are shown in Table 1. There were statistically significant differences in HGB and DD characteristics between the two groups (*P* < 0.05, Table 1). As shown in Fig. S4, the absolute values of the correlation coefficients among the seven selected features were all less than 0.3.

**Table 1:** Comparison of baseline demographic and laboratory characteristics between deep vein thrombosis (DVT) patients and non-DVT control subjects. This table summarizes the baseline data of 231 enrolled subjects, including 159 patients in the DVT group and 72 subjects in the non-DVT control group. Continuous variables are presented as median (±standard deviation, SD), and categorical variables are presented as number (percentage). The Wilcoxon rank sum test was used for intergroup comparison of continuous variables, and Pearson’s Chi-squared test was used for categorical variables; the *p*-value for each characteristic corresponds to the intergroup comparison result. The “Unknown” row indicates the number of cases with missing values for the corresponding indicator.

**Characteristic**	**Overall**, *N* = 231	**Yes**, *N* = 159	**No**, *N* = 72	***p*** **-value** ^1^
Age, Median (±SD)	61 (±17.35)	62 (±18.11)	60.50 (±15.67)	0.8
Gender, n (%)				0.12
Male	127 (55%)	82 (52%)	45 (63%)	
Female	104 (45%)	77 (48%)	27 (38%)	
WBC, Median (±SD)	7.91(±4.37)	7.61 (±3.52)	8.25 (±5.79)	0.6
Unknown	4	2	2	
HGB, Median (±SD)	122 (±25.41)	117 (±25.74)	134 (±21.26)	<0.001
Unknown	4	2	2	
PLT, Median (±SD)	243 (±98.88)	247 (±105.15)	230.50 (±81.10)	0.2
Unknown	4	2	2	
FIB, Median (±SD)	3.37 (±1.43)	3.35 (±1.36)	3.53 (±1.55)	0.3
Unknown	1	1	0	
DD, Median (±SD)	2.48 (±20.00)	4.99 (±23.04)	0.56 (±2.95)	<0.001
Unknown	11	11	0	
PT2, Median (±SD)	100.70 (±25.24)	98 (±23.98)	103.55(±27.55)	0.029
GLU, Median (±SD)	6.19 (±4.17)	5.97 (±3.51)	7.14 (±5.27)	0.055
Unknown	38	25	13	

**Notes.**

1 Wilcoxon rank sum test; Pearson’s Chi-squared test.

Abbreviations DVT deep vein thrombosis WBC white blood cell HGB hemoglobin PLT platelet FIB fibrinogen DD D-dimer PT prothrombin time GLU fasting blood glucose

### Model building and evaluation

**Figure 2:**
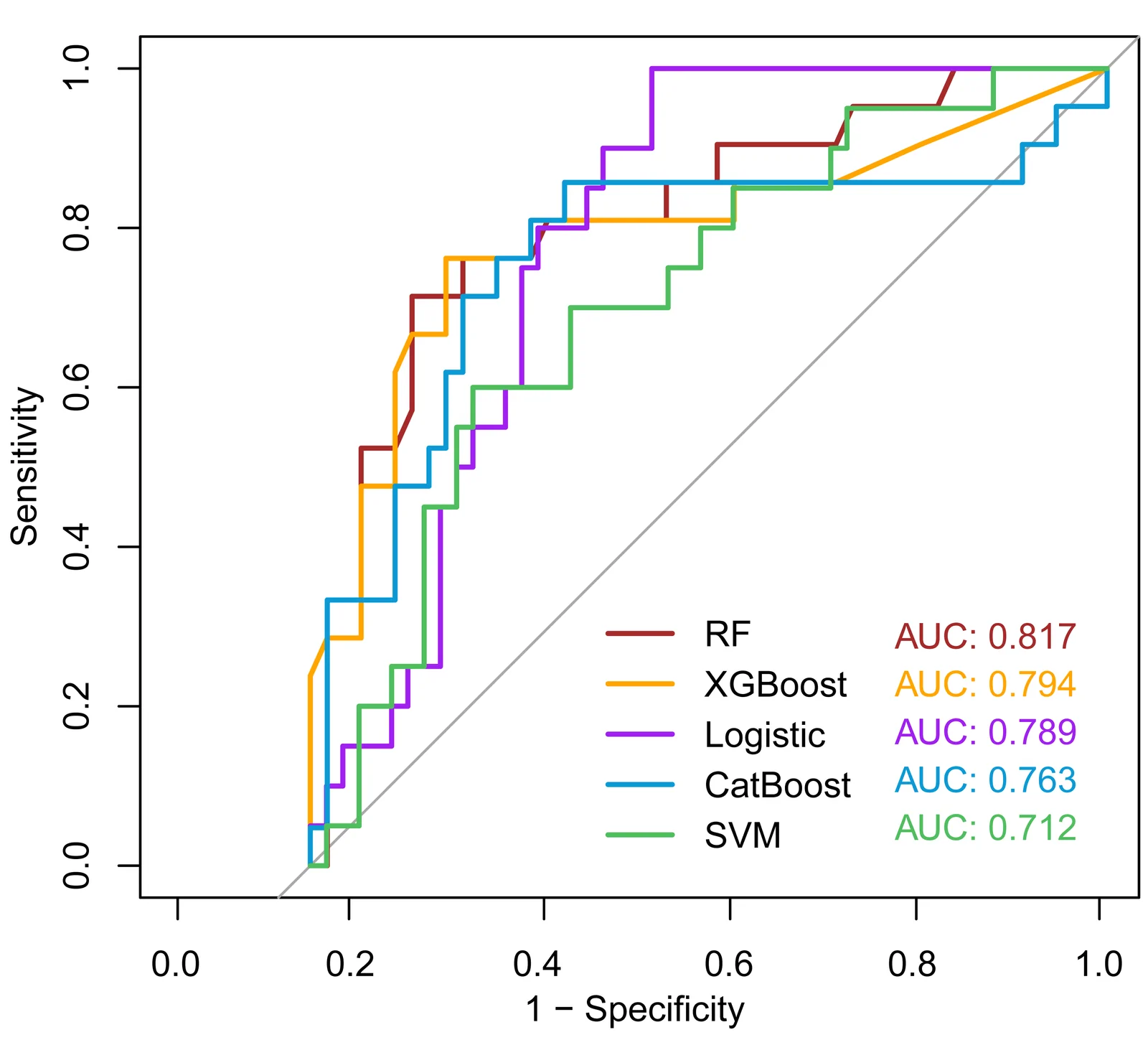
Receiver operating characteristic (ROC) curves of five machine learning models for deep vein thrombosis prediction from a representative single run on the test set. This figure presents the receiver operating characteristic (ROC) curves of five machine learning models for predicting the risk of deep vein thrombosis (DVT), derived from a single representative run on the independent test set. The *x*-axis represents 1 − Specificity, and the *y*-axis represents Sensitivity. The area under the ROC curve (AUC) was calculated to quantify the discriminative performance of each model. The AUC values for each model are as follows: Random Forest (RF) 0.817, Extreme Gradient Boosting (XGBoost) 0.794, Logistic Regression 0.789, Categorical Boosting (CatBoost) 0.763, and Support Vector Machine (SVM) 0.712.

Within the training data set, the XGBoost, LR, RF, CatBoost and SVM models were established, and model performance was evaluated using 5-fold cross-validation repeated 1,000 times. The model performance was evaluated using four metrics: accuracy, recall, F1 score, and AUC. Figure 2 presents the ROC curves and AUC values from a single representative model run on the test set, whereas Table 2 summarizes the model performance across 1000 repeated runs and reports the mean AUC with 95% confidence intervals. The testing data set obtained AUCs of 0.8490, 0.8553, 0.8744, 0.8622, and 0.7429, respectively (Table 2). Comparatively, RF had the highest predictive performance among the four models (AUC = 0.8744, 95% CI [0.7877–0.9448]), whereas SVM had the poorest generalization ability (AUC = 0.7429, 95% CI [0.6315–0.8501]). RF had the highest score among F1 scores, accuracy, recall and AUC (Table 2). ROC dotted line chart is the result of the testing set, RF, XGBoost, Logistic model, Catboost, the area under the roc curve of SVM are respectively 0.817, 0.794, 0.789, 0.763, 0.712 (Fig. 2). as a result, the result of the training set and testing set are said RF optimal model.

**Table 2:** Performance summary of machine learning models. This table provides a comparative evaluation of five machine learning models—XGBoost, CatBoost, Random Forest (RF), Logistic Regression, and Support Vector Machine (SVM)—in predicting a binary outcome, based on key performance metrics. Each model is assessed using accuracy, precision, recall, F1-score, and area under the receiver operating characteristic curve (AUC), with all metrics reported alongside their 95% confidence intervals (95% CI) to quantify uncertainty. The results highlight RF as the top-performing model, achieving the highest accuracy (0.8235), precision (0.8571), recall (0.9149), F1-score (0.8800), and AUC (0.8744), though XGBoost and CatBoost also demonstrate competitive performance.

**model**	**Accuracy (95% CI)**	**Precision (95% CI)**	**Recall (95% CI)**	**F1 (95% CI)**	**AUC (95% CI)**
XGBoost	0.8088 (0.7206–0.8824)	0.8367 (0.7627–0.9130)	0.9149 (0.7872–0.9787)	0.8660 (0.8090–0.9167)	0.8490 (0.7452–0.9316)
CatBoost	0.8088 (0.7353–0.8824)	0.8600 (0.7925–0.9286)	0.8723 (0.7447–0.9787)	0.8660 (0.8000–0.9215)	0.8622 (0.7619–0.9382)
RF	0.8235 (0.7353–0.8971)	0.8571 (0.7857–0.9302)	0.9149 (0.7872–0.9787)	0.8800 (0.8132–0.9278)	0.8744 (0.7877–0.9448)
Logistic	0.7826 (0.6957–0.8696)	0.7925 (0.6842–0.8852)	0.9388 (0.8444–1.0000)	0.8571 (0.7885–0.9143)	0.8553 (0.7679–0.9253)
SVM	0.7246 (0.6377–0.8116)	0.7377 (0.6290–0.8394)	0.9535 (0.8542–1.0000)	0.8270 (0.7573–0.8850)	0.7429 (0.6315–0.8501)

**Notes.**

XGBoost, Extreme Gradient Boosting; CatBoost, Categorical Boosting; RF, Random Forest; Logistic, Logistic Regression; SVM, Support Vector Machine.

### Explanation of RF model with the SHAP method

**Figure 3:**
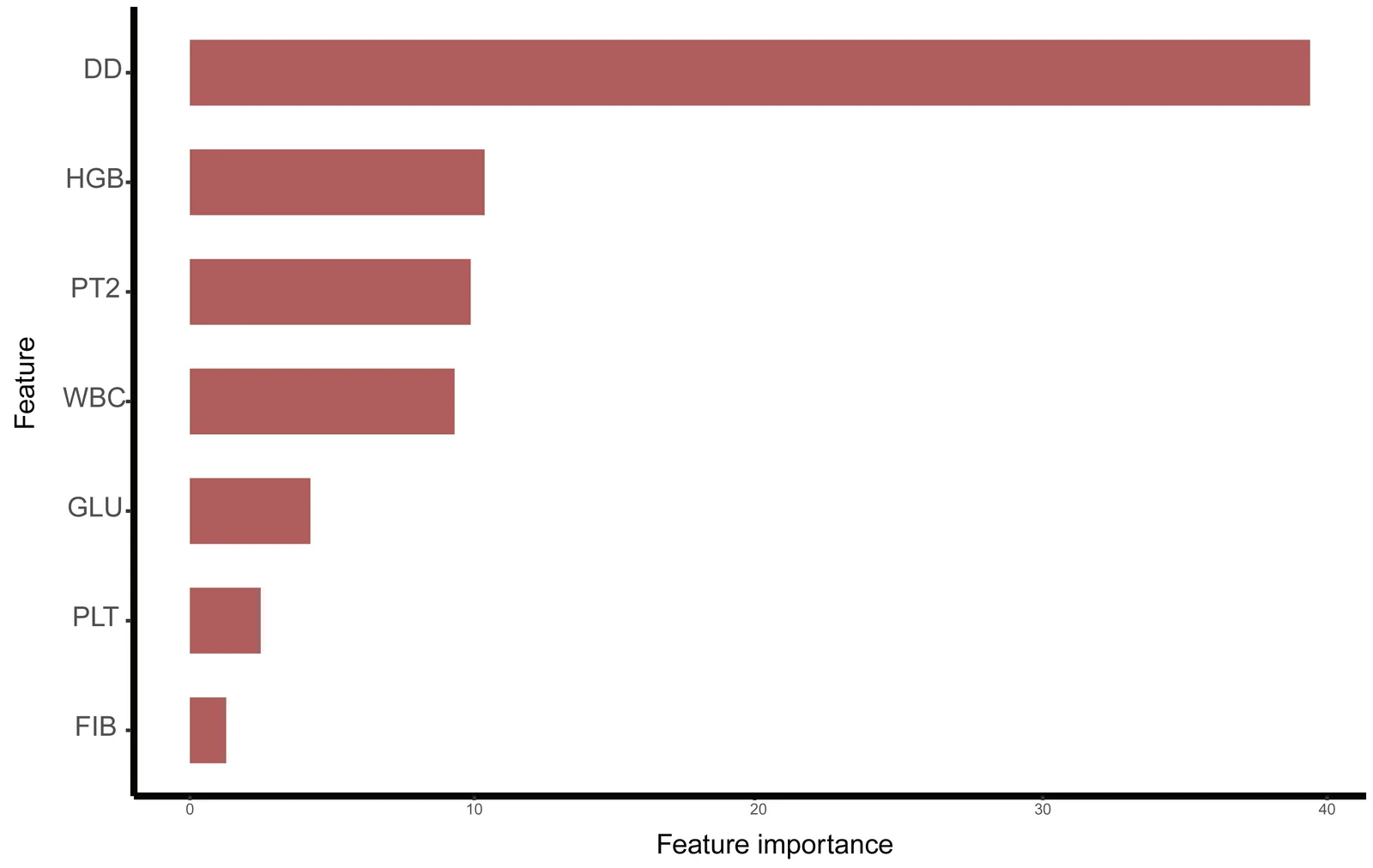
Feature importance diagram based on RF models.

**Figure 4:**
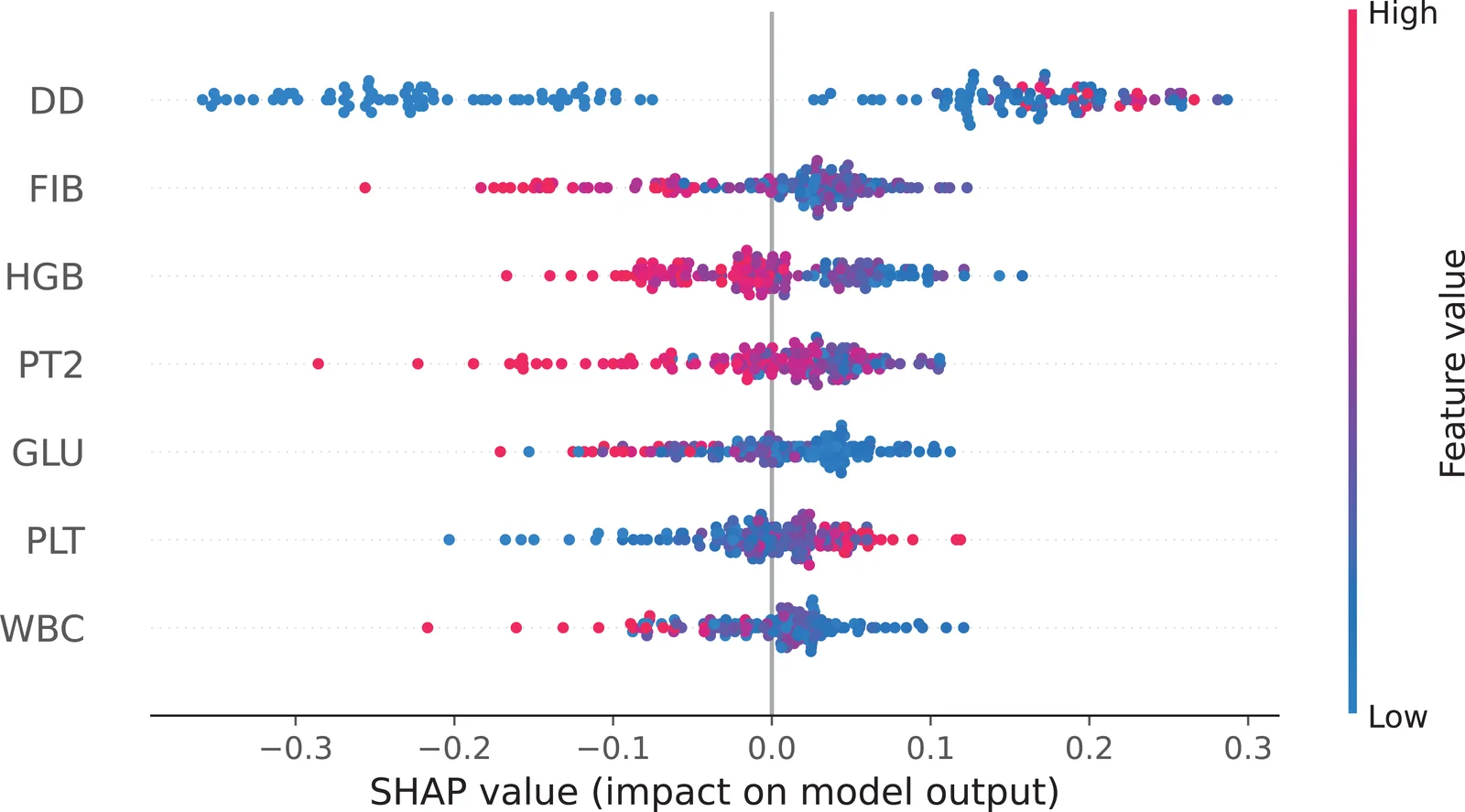
Summary diagram of SHAP based on RF model.

**Figure 5:**
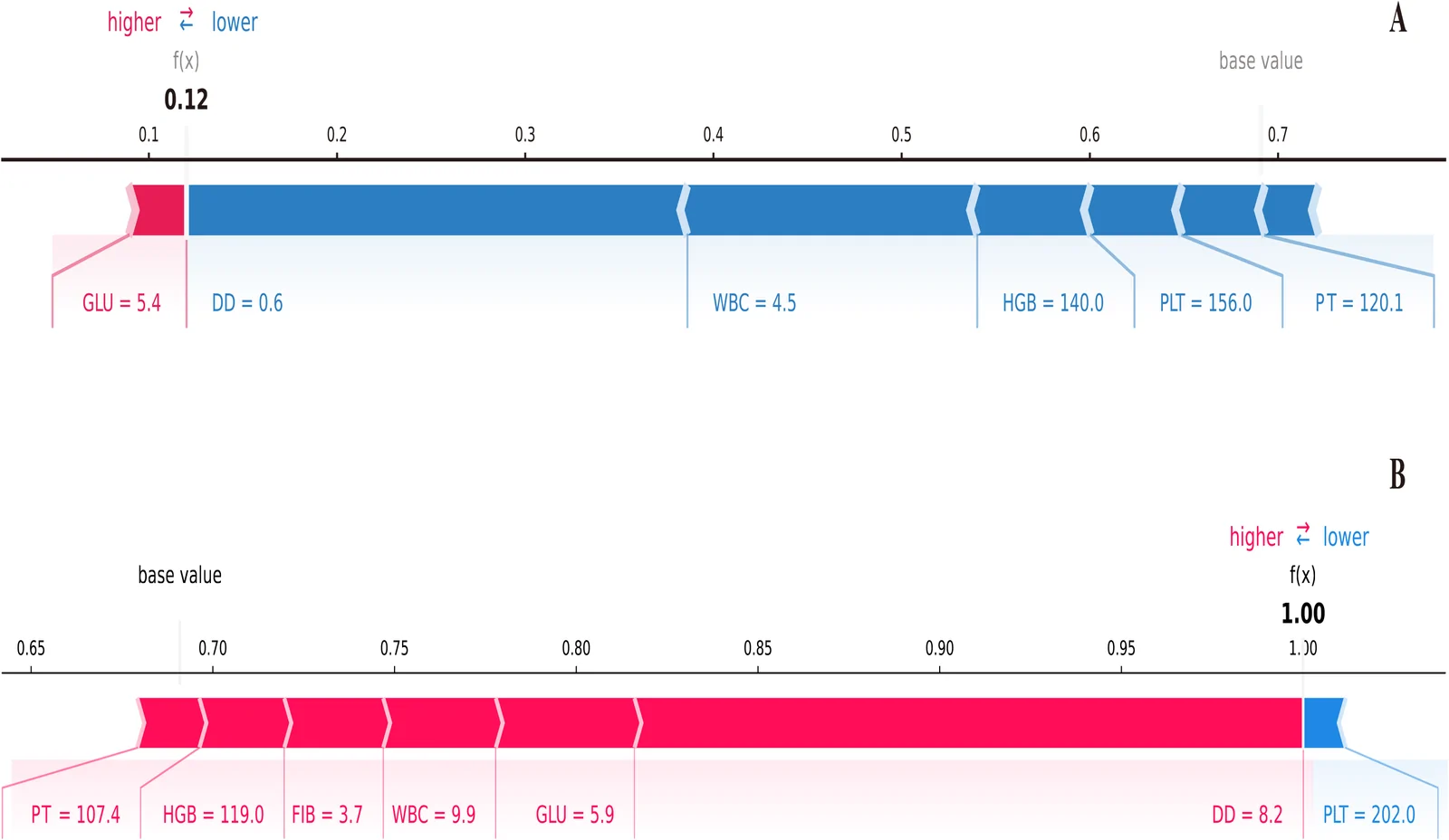
Corresponds to the SHAP example of the RF model predicting the risk score of DVT occurrence.

SHAP values were used to obtain direction and magnitude of each predictor variable to the outcome predicted by the RF model. The variable importance plot lists the most significant variables in a descending order (Fig. 3). DD had the highest mean absolute SHAP value, indicating the strongest overall contribution to model predictions, followed by the HGB factor, PT, WBC, GLU, PLT and FIB. Furthermore, to detect the positive and negative relationships of the features with the target result, SHAP values were applied to uncover the DVT risk factors. As presented in Fig. 4, the horizontal location shows whether the effect of that value is associated with a higher or lower prediction and the color shows whether that variable is high (in red) or low (in blue) for that observation; we can see that increases in the average DD has a positive impact and push the prediction toward DVT. Figure 5 illustrates high-risk and low-risk patients according to the RF model, detailing the contribution and influence direction of each feature. Figure 5A illustrates a representative patient with a low predicted risk of DVT. The SHAP values show how each observed clinical feature contributes to the final prediction score relative to the baseline value; although GLU pushes the prediction toward higher risk, the combined effects of the remaining variables shift the overall prediction toward lower risk. In Fig. 5B, the f(x) value of 1.00 indicates a high risk of DVT, with significant red features (DD, PLT, WBC) contributing to this risk. This analysis highlights that increased DD levels correlate positively with DVT, underscoring its significant impact.

## Discussion

This study presents the development and internal validation of a ML model designed for the early prediction of deep vein thrombosis (DVT) in a hospitalized patient population. The primary contribution is the creation of a predictive tool based on a parsimonious set of seven routinely available laboratory indicators. This model was derived from a retrospective analysis of 231 patients admitted to the Fifth Affiliated Hospital of Southern Medical University between January 2017 and June 2024, of whom 159 had a DVT occurrence and 72 did not. The methodological approach employed LASSO regression to identify this 7-variable predictive signature from a larger set of potential features, which include HGB, PLT, WBC, FIB, PT, DD, and GLU. The reliance on these common biomarkers underscores the model’s potential for low-cost, high-efficiency integration into standard clinical workflows (Chen et al., 2025). A comparative analysis of five distinct ML algorithms—RF, CatBoost, XGBoost, LR and SVM—was conducted. Our ML models, particularly RF and XGBoost, demonstrated superior performance in predicting DVT compared to traditional LR. The RF model achieved the highest AUC of 0.817, outperforming previos studies (Wang et al., 2022b). This underscores the potential of ML in clinical DVT risk assessment. RF outperformed advanced gradient boosting methods like XGBoost (AUC = 0.849) and CatBoost (AUC = 0.862) is noteworthy. A large-scale 2023 study of over 109,000 critically ill patients in the eICU database also identified Random Forest as the most effective and reliable model, achieving an AUC of 0.938 and outperforming both XGBoost and SVM, which was corresponding to our results (Guan et al., 2023). Our model’s performance is similar with several externally validated models from 2025, which reported AUCs of 0.840 and 0.810 (Chen et al., 2025; Sun et al., 2025). It also competes favorably with the 0.864 prospective AUC achieved by the “PE-Mind” deep learning model (Tian et al., 2025). The fact that our parsimonious, interpretable RF model holds its own in this elite company strongly indicates a valid and promising architecture

For patients diagnosed with DVT, we found significant differences in HGB and DD levels between patient groups. Previous findings have also revealed that HGB and DD are among the possible risk factors (Xiong, Li & Cheng, 2023; Zhang, Wu & Cheng, 2022), highlighting the importance of addressing anemia before surgery to reduce perioperative complications including DVT. A study analyzing features for VTE-related risk similarly identified a negative correlation for HGB, where lower HGB (anemia) was associated with higher risk (Al Raizah et al., 2024). Anemia may represent a state of chronic systemic stress or hypoxia, which is known to activate pro-thrombotic pathways (Schulman et al., 2024). DD is a biomarker for fibrinolysis and coagulation activation and proves valuable in assessing VTE risk (Biciusca et al., 2024; Parpia et al., 2020; Weitz, Fredenburgh & Eikelboom, 2017). Its elevation in the blood is a direct and sensitive biomarker signifying that both coagulation (clot formation) and subsequent fibrinolysis (clot breakdown) are actively occurring (Fu et al., 2024). Feature importance and SHAP analyses identified the contribution of key DVT risk factors. DD emerged as the most critical indicator, with its elevation significantly increasing DVT risk (Han et al., 2023; Tong et al., 2024). Other important factors included HGB, PLT, WBC, FIB, PT, GLU. The inclusion of both GLU and FIB is not redundant, which captures a critical and dangerous synergistic interaction. Chronic hyperglycemia (elevated GLU) drives a non-enzymatic process called glycation (Borghi et al., 2025). Fibrinogen is a primary target of this glycation.

The SHAP interpreter provided a visual tool for personalized DVT risk assessment, offering insights into the weight of contributing factors for individual patients (Kuan et al., 2025). This methodology moves our model beyond the limitations of traditional, static risk scores like the Wells or Padua scores, which stratify risk at a population level (Chen et al., 2024). This approach advances beyond traditional statistical methods by enabling patient-specific risk evaluation (Fu et al., 2024; Shibata et al., 2023; Zeng et al., 2023). However, our study has limitations. The sample size may not fully capture DVT complexity, and the single-center of the data may limit result generalizability. Future research should expand sample sizes, incorporate more diverse data sources, and consider additional features to enhance model predictive efficiency. Developing an automated, personalized DVT risk assessment system could significantly improve clinical decision-making and patient outcomes.

## Conclusion

Our ML model approach offers a promising tool for DVT risk prediction, outperforming traditional methods. By identifying key risk factors and enabling personalized risk assessment, this model has the potential to significantly improve DVT prevention and management strategies in clinical practice.

## Supplemental Information

10.7717/peerj.21524/supp-1 Supplemental Information 1 missing data distribution

10.7717/peerj.21524/supp-2 Supplemental Information 2 Variable screening based on Lasso regression The tuning hyperparameter (*λ*) in the LASSO model is selected for 5-fold cross-validation by the minimum criterion. The dashed line on the right represents the optimal value based on the minimum criterion and its 1 standard error (1-SE )

10.7717/peerj.21524/supp-3 Supplemental Information 3 The path distribution of regression coefficients of log (*λ*) sequence in Lasso regression model was selected by cross-validation method

10.7717/peerj.21524/supp-4 Supplemental Information 4 Correlation coefficient heat map

10.7717/peerj.21524/supp-5 Supplemental Information 5 Raw data codebook Sex: Male = man, Female = woman. Age: years. DVT: 0 = no deep-vein thrombosis, 1 = DVT diagnosed. RBC: erythrocyte count (10^12^/L).WBC: leukocyte count (10/L).HGB: haemoglobin (g/L).PLT: platelet count (10/L).FDP: fibrinogen-degradation products (mg/L).APTT: activated partial thromboplastin time (s).FIB: fibrinogen (g/L).PT: prothrombin time (s).PTR: prothrombin ratio.DD: D-dimer (mg/L FEU).TT: thrombin time (s).INR: international normalised ratio.PT2: second prothrombin-time measurement (s).CREA: creatinine (µmol/L).GLU: glucose (mmol/L). All continuous variables: numeric, NA = not available.

10.7717/peerj.21524/supp-6 Supplemental Information 6 Summary of patient laboratory values The patient’s blood test results include the following parameters: Deep Vein Thrombosis (DVT) status, Red Blood Cell count (RBC), White Blood Cell count (WBC), Hemoglobin (HGB), Platelet count (PLT), Fibrinogen Degradation Products (FDP), Activated Partial Thromboplastin Time (APTT), Fibrinogen (FIB), Prothrombin Time (PT), Prothrombin Time Ratio (PTR), D-Dimer (DD), Thrombin Time (TT), International Normalized Ratio (INR), second Prothrombin Time measurement (PT2), Creatinine (CREA), and Glucose (GLU).

## Additional Information and Declarations

### Data Availability

The following information was supplied regarding data availability: The raw data is available in the Supplemental File.
