# Supplementary figures and images for: A machine learning-based risk prediction model for Hospitalized patients with deep vein thrombosis
Source: PeerJ. 2026 Jul 30;14:e21524. doi: 10.7717/peerj.21524 (PMC13429103; doi:10.7717/peerj.21524)

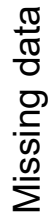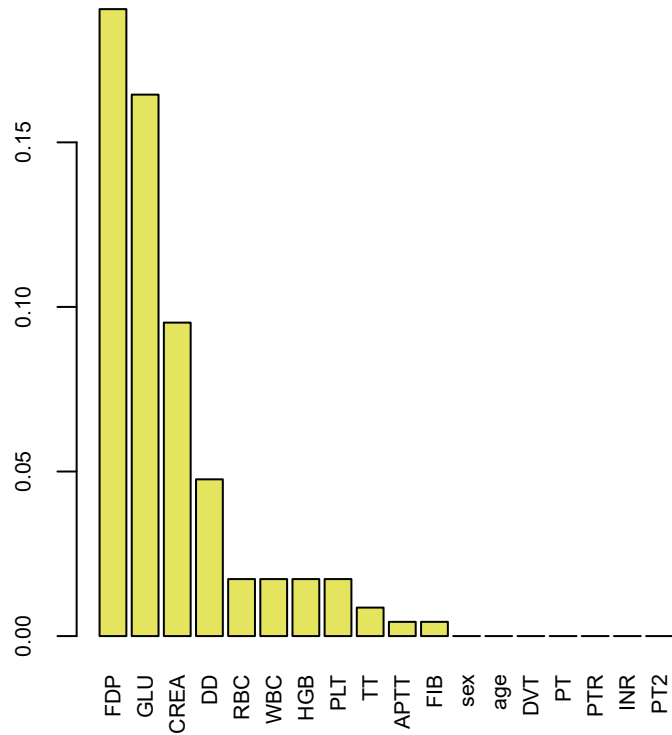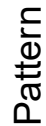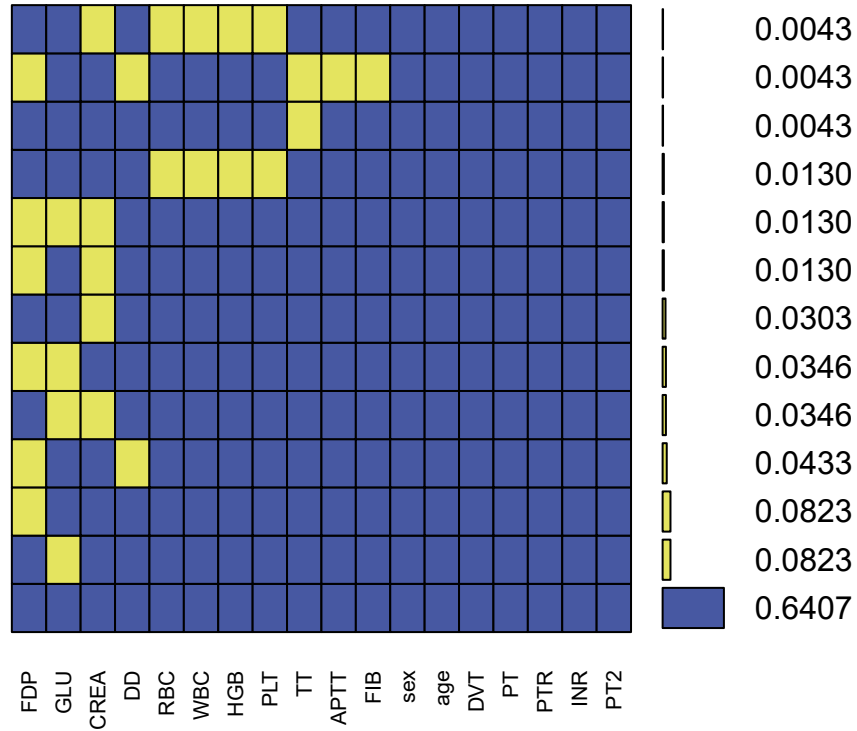

Supplement: Supplemental Information 1 [file peerj-14-21524-s001.pdf]

Binomial Deviance

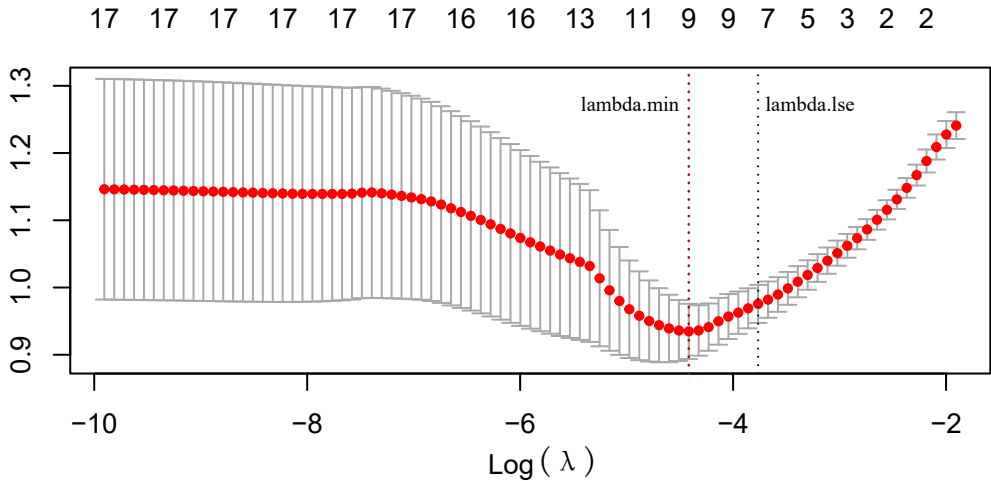

Supplement: Supplemental Information 2 — The tuning hyperparameter (λ) in the LASSO model is selected for 5-fold cross-validation by the minimum criterion. The dashed line on the right represents the optimal value based on the minimum criterion and its 1 standard error (1-SE ) [file peerj-14-21524-s002.pdf]

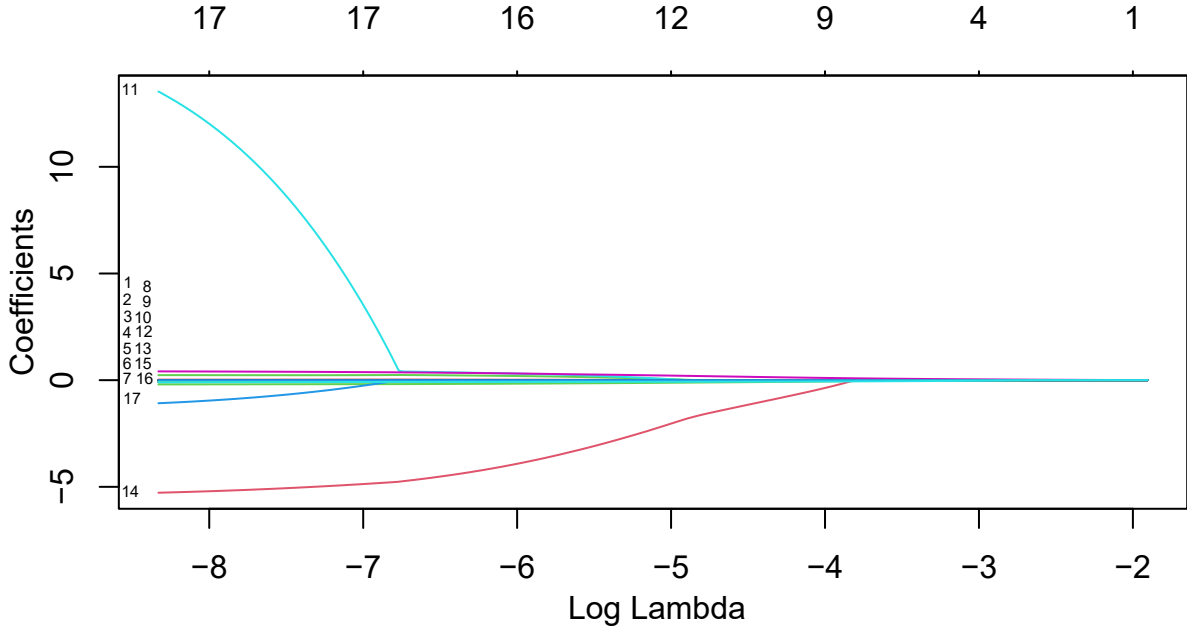

Supplement: Supplemental Information 3 [file peerj-14-21524-s003.pdf]

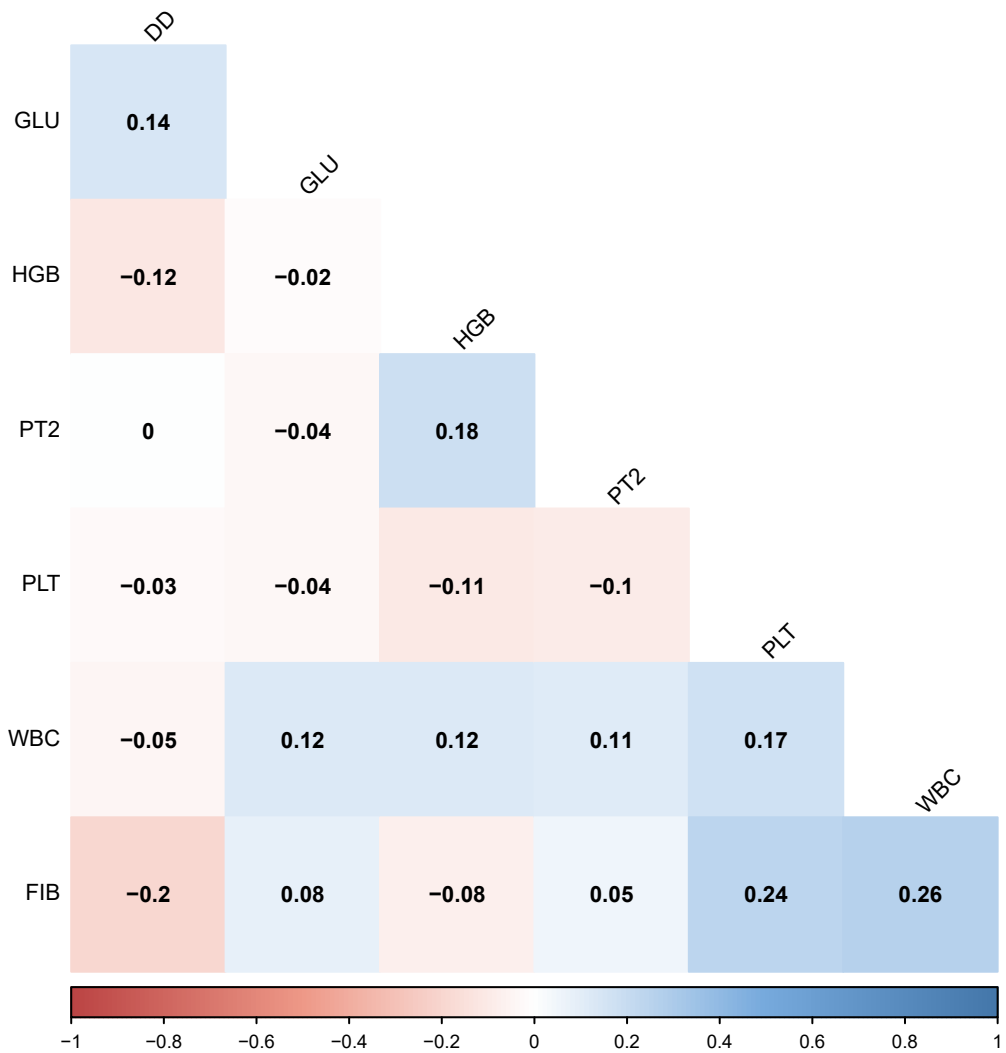

Supplement: Supplemental Information 4 [file peerj-14-21524-s004.pdf]
